# Building Noncommunicable Disease Workforce Capacity Through Field Epidemiology Training Programs: Experience From India, 2018–2021

**DOI:** 10.5888/pcd19.220208

**Published:** 2022-12-08

**Authors:** Archana Ramalingam, Mohankumar Raju, Parasuraman Ganeshkumar, Rajesh Yadav, Sukarma Tanwar, Manikandanesan Sakthivel, Qaiser Mukhtar, Prabhdeep Kaur

**Affiliations:** 1 National Institute of Epidemiology, Indian Council of Medical Research, Chennai, Tamil Nadu, India; 2 Centers for Disease Control and Prevention, New Delhi, Delhi, India; 3 Centers for Disease Control and Prevention, Atlanta, Georgia, USA

## Rationale

By 2003, India had started to shift from a high burden of communicable diseases to noncommunicable diseases (NCDs). By 2019, NCDs accounted for two-thirds of all deaths in India (1,2). However, the epidemiologic transition of growth of NCD burden was not uniform among all states. Thus, state-specific policy decisions and program strategies are required to address the growing NCD burden.

In response to rising NCD prevalence, India launched the National Programme for Prevention and Control of Cancer, Diabetes, Cardiovascular Diseases, and Stroke (NPCDCS) in 2010 to cover all districts in India (3). The program focused on prevention, screening, diagnosis, and management of hypertension, diabetes, cardiovascular disease, and cancer. Program implementation in the states has faced challenges because of a poorly designed monitoring system, interruptions in drug supply, unreliable access to diagnostics, and poor financial planning. A skilled public health workforce at the state and district levels is required to monitor, analyze, and interpret program data to identify key challenges and implement evidence-based strategies to address the challenges (4).

An approach that India is taking to strengthen the quality of the nation’s public health systems relies on training the public health workforce through Field Epidemiology Training Programs (FETPs). FETPs, rooted in the concept of “learning by doing” under mentorship, impart key epidemiologic skills to the frontline public health workforce (epidemiologists, surveillance and program officers), providing them with the skills to conduct field investigations and take appropriate public health actions (5). As FETP trainees analyze program data, evaluate surveillance systems, and perform epidemiologic investigations, they develop critical thinking and problem-solving skills (6,7). Worldwide, in public health emergencies such as the COVID-19 pandemic and Ebola virus disease outbreaks, FETPs have helped build resilient health systems (8).

The initial focus of FETPs in India has been on investigating infectious diseases. However, with the ongoing epidemiologic transition and the growing NCD burden, India needs to build the capacity of public health professionals already working in the field to address NCDs and their risk factors at national and subnational levels. We describe India’s efforts to build public health workforce competencies to respond to the threats of NCDs through the creation of an NCD-specific track in their FETPs.

## Establishing the NCD Track of FETP in India

The Indian Council of Medical Research’s National Institute of Epidemiology, Chennai (ICMR-NIE) has nearly 2 decades of experience in conducting full-time master’s-level programs built on the FETP core competencies. In 2012, a 2-year advanced FETP, the India Epidemic Intelligence Service (India EIS) program, was started at National Centres for Disease Control in India in collaboration with US Centers for Disease Control and Prevention (US-CDC). The goal is to have 1 trained field epidemiologist in every district (~770 districts), by selecting one among the surveillance officers, program managers, and epidemiologists in the district. In 2016, to meet the country’s epidemiologists’ training needs, the Ministry of Health and Family Welfare of the Government of India expanded the network of institutions offering the India EIS; ICMR-NIE was selected as one of the hubs for the program. Understanding the need for NCD-specific training, ICMR-NIE, in collaboration with CDC-India, launched a separate track of FETP for NCDs called FETP-NCD. The FETP-NCD track had 2 tiers; the FETP-NCD advanced (2-year) started in 2018 and FETP-NCD intermediate (1-year) started in 2021. The 2 tiers were started keeping in view the differing training needs of public health professionals who work in leadership positions and those working as midlevel managers.

### Collaborator consultations

Before the program’s launch, the course coordinators held discussions with key collaborators to understand training needs and best practices. The participants of the meetings included officials from state public health departments (India), FETP course coordinators from other countries (Thailand, Ethiopia, China), public health experts from US-CDC and CDC-India, and FETP alumni and mentors. Inputs from these meetings contributed to the basic structure of the advanced FETP-NCD program, mentorship requirements, and recruitment strategy. The inputs also highlighted the need to add an intermediate tier targeting the competency needs of midlevel managers.

### Recruitment of trainees

The advanced FETP-NCD program admitted medical professionals who worked in leadership positions at the national, state, or district levels of the NCD program or agencies supporting NCD programs. The intermediate program admitted program managers who worked with the NCD program (medical degree not required). The candidates interested in the program applied when the admissions were open. The candidates who satisfied the eligibility criteria were interviewed. The final selection was based on a score that accounted for their additional educational qualifications, public health experience, and performance in an interview. The advanced FETP-NCD admits a maximum of 15 candidates each year and the intermediate tier admits 25 candidates.

### Basic structure of the FETP-NCD programs

The FETP-NCD programs of the ICMR-NIE are part-time in-service training (compared with full-time regular India EIS). Participants work in their respective state or district NCD placement sites without being assigned to ICMR-NIE. The basic structure and core activities of learning (competencies) of the FETP-NCD curricula (Table) align with FETPs around the globe.

**Table:** Structure of the Advanced and Intermediate Field Epidemiology Training Programs (FETPs) for Noncommunicable Diseases (NCDs), India

Domain	Advanced FETP	Intermediate FETP
**Targeted learners**	National and state level NCD nodal officers (physicians)	District NCD nodal officers and program managers (physicians and allied public health professionals)
**Duration**	2 years: • Classroom training (12–14 weeks) • Field posting (72–74 weeks)	1 year: • Classroom training (8–10 weeks) • Field posting (38–40 weeks)
**Mode of training**	• In-person workshop sessions • Webinars • Small group training at field posting sites	• In-person workshop sessions • Webinars • Small group training at field posting sites
**Teaching–learning methods**	• Lectures • Group discussion • Case studies • Hands-on training (Microsoft Corporation applications, Epi Info version 7.2) • Fieldwork	• Lectures • Group discussion • Case studies • Hands-on training (Microsoft Corporation applications, Epi Info version 7.2) • Fieldwork
**Mentor: mentee ratio**	• 1:3	• 1:5
**Core activities of learning (no. required for graduation)**	• Secondary data analysis of program data (2) • Field investigation (1) • Planned analytical epidemiology study (1) • Program evaluation (1) • Abstract (1) • Manuscript (1) • Oral or poster presentation at a scientific conference (1)	• Secondary data analysis of program data (1) • Field investigation (1) • Group work: analytical epidemiology study (1) • Group work: program evaluation (1) • Abstract (1) • Oral or poster presentation at a scientific conference (1)

### Building a pool of dedicated mentors

Because mentoring is critical to the success of FETP-NCD, faculty mentors (1:3 mentor:mentee ratio for advanced and 1:5 for intermediate) were chosen based on their experience in field epidemiology, mentoring expertise, and interpersonal skills. FETP-NCD mentors have included scientists from NIE who work in NCDs, previous FETP graduates, and public health experts from CDC-India, multinational nongovernment organizations, and CDC-India implementing the partner South Asia Field Epidemiology and Technology Network (SAFETYNET). In addition, FETP-NCD faculty regularly participated in mentorship training and developed advancements in teaching and learning techniques and interpersonal skills. Senior mentors (those with 5 or more years of mentorship experience) support junior mentors as co-mentors.

### Progress of the advanced FETP-NCD

We initiated FETP-NCD advanced in 2018 with 5 trainees. Two of the 5 trainees graduated in 2020; the other 3 did not complete the program because of competing work commitments related to the COVID-19 response. The second cohort began in November 2019 with 8 trainees. Because of the pandemic, classroom contact sessions were hybrid, and we provided an extension to the second cohort (expected graduation in December 2022). The third cohort of 15 trainees was inducted into the advanced FETP-NCD in September 2021 and are due to graduate in September 2023.

In the initial year, program staff adapted FETP training materials to focus on NCD-related topics, including NCD-based case studies. The curriculum had a separate module on NCDs focusing on epidemiology of cardiovascular diseases, diabetes, and cancers; NCD risk factor surveillance; NCD program data analysis; and preventive strategies for NCDs.

All projects for completing the core activities of learning (Table) were done in priority areas of the NCD program in India. A few examples include 1) analysis of secondary data from the NCD program to assess the treatment outcomes (blood pressure control status) of hypertension patients, 2) field investigation to assess the reasons for missed visits by hypertension patients, 3) evaluation of the diabetes program in Kerala to understand the gaps in program implementation, and 4) an advanced epidemiology study to assess the compliance to hypertension treatment protocol by treating physicians. Each of the above-mentioned projects done as part of the core activities of learning were vital in providing information for action to improve implementation of the national NCD program.

Cardiovascular diseases are the leading causes of deaths in India. One of the key targets of India’s national NCD program is to reduce the premature mortality attributable to cardiovascular diseases by 25% by 2025, in line with the global voluntary NCD targets. Since hypertension control is critical to preventing adverse cardiovascular events, most of the trainees’ projects were focused on hypertension. Two of the first cohort graduates received small, competitive grants from TEPHINET (Training Programs in Epidemiology and Public Health Interventions Network), a global network of FETPs. Since clinical inertia is one of the key factors that affect blood pressure control in the population, one of the small grant projects focused on assessing the compliance of physicians in primary and secondary care health centers to hypertension treatment protocols. The study found that nearly three-fifths of the prescriptions by physicians adhered to treatment protocol. After the study concluded, refresher trainings were done for the physicians, emphasizing the need to adhere to the treatment protocol. The second small grant project focused on forecasting, procurement process, and availability of protocol-based antihypertensive drugs at public health facilities in 4 states (Punjab, Madhya Pradesh, Telangana, and Maharashtra) of India between June 2019 and May 2020. The study found that the drug forecasting tool (provided as part of the India Hypertension Control Initiative) helped improve drug availability over time. It also found a gap in the knowledge of district level NCD nodal officers about the drug forecasting process, which was later addressed through refresher trainings. These examples demonstrate that the FETP trainees’ projects generate vital information that is used for planning interventions to improve the NCD program. In addition, during COVID-19, the FETP-NCD trainees also led innovations to ensure continuum of care for hypertension patients, including establishing door-to-door drug delivery systems and designing and implementing telehealth services (9,10). The FETP trainees also routinely disseminate findings from the projects to the state ministries of health for necessary public health action. They also presented some of the projects at conferences to share the best practices, sometimes winning the best paper presentations.

### Progress of the intermediate FETP-NCD

We initiated the intermediate FETP-NCD at ICMR-NIE in October 2021. The first cohort of the intermediate FETP-NCD started with 22 trainees from 10 different states in India. The trainees conducted NCD program data analysis (screening, diagnosis, and treatment) at the district and state levels, providing critical information on hypertension or diabetes control rates.

## Challenges

Despite rapid expansion, high demand, and early success, both FETP-NCD programs have numerous challenges. Lack of buy-in from state health departments because of lack of prioritization of NCDs remains a challenge. The trained FETP alumni are often underused (assigned additional clinical responsibilities rather than public health–related duties). The absence of defined career pathways following program completion deters candidates from applying. Given the in-service training model, difficulties balancing work-related commitments while fulfilling rigorous training requirements lead to dropouts. Identifying, developing, and retaining mentors for the FETP-NCD is another major challenge. Finally, innovative solutions are required to reduce the administrative burden of program expansion.

## Way Forward

ICMR-NIE is initiating state-specific intermediate FETP-NCD with the state health leadership in Chhattisgarh and Odisha to train 1 person in NCDs in every district. In this state-specific model, ICMR-NIE plans to enroll state-nominated trainees in each state and conduct in-person training sessions and mentorship in the respective states with support from full-time state-based mentors. The intermediate FETP-NCD, tailored to state-specific needs, allows the state health department to take ownership of the program and identify the training needs and priorities for field projects. Efforts are under way to mitigate workload by aligning the core activities of learning with the on-the-job profile of the trainees and increasing acceptability by preparing for TEPHINET accreditation of the FETP-NCD. In addition, India needs to establish networks for FETP alumni and faculty for experience sharing, mutual learning, and increasing the available pool of mentors. Beyond this, to ensure sustainability and scale-up, policy makers at the state and central ministries of health need to allocate sufficient funds for mentor trainings. Finally, digital innovations such as learning management software currently being piloted in India will improve the delivery of FETPs and reduce administrative burden.

In light of India’s large population and 770 districts, commitment from leadership, funding, and ownership from the Ministry of Health and Family Welfare and states will be required to scale and sustain advanced and intermediate FETP-NCD. Advanced FETP-NCD is needed to develop skilled public health leaders at the national, regional, and state levels. Intermediate FETP-NCD is expandable, can be embedded in the state health systems, and is more suited for the competency needs of the state and district-level public health workforce. FETP-NCD programs will better equip India with a skilled workforce to address the increasing NCD burden and serve as a model for other FETPs.
